# A two-step mechanism for RIG-I activation by influenza virus mvRNAs

**DOI:** 10.1126/sciadv.adw8034

**Published:** 2025-08-08

**Authors:** Emmanuelle Pitré, Karishma Bisht, Kaleigh A. Remick, Amir Ghorbani, Jonathan W. Yewdell, Elizaveta Elshina, Aartjan J.W. te Velthuis

**Affiliations:** ^1^ Lewis Thomas Laboratory, Department of Molecular Biology, Princeton University, Princeton, NJ 08544, USA.; ^2^ Department of Pathology, Addenbrooke’s Hospital, University of Cambridge, Cambridge CB2 2QQ, UK.; ^3^ Cellular Biology Section, Laboratory of Viral Diseases, National Institute of Allergy and Infectious Diseases, Bethesda, MD 20892, USA.

## Abstract

Influenza A virus (IAV) noncanonical RNAs are bound by retinoic acid–inducible gene I (RIG-I). However, innate immune activation is infrequent and it is not understood why noncanonical IAV RNAs activate RIG-I in a sequence- or RNA structure–dependent manner. We hypothesized that multiple events need to occur before IAV RNA synthesis activates RIG-I and investigated whether RIG-I activation is stimulated by the noncanonical or aberrant transcription of mini viral RNAs (mvRNA), an RNA that is overexpressed in highly pathogenic IAV infections. We find that mvRNAs can cause noncanonical transcription termination through a truncated 5′ polyadenylation signal or a 5′ transient RNA structure that interrupts polyadenylation. The resulting capped complementary RNAs stimulate the release of an mvRNA and complement RIG-I activation in trans. Overall, our findings indicate that sequential rounds of noncanonical or aberrant viral replication and transcription are needed for innate immune signaling by IAV RNA synthesis.

## Teaser

Sequential rounds of noncanonical influenza virus replication and transcription activate RIG-I.

## INTRODUCTION

Influenza A viruses (IAVs) cause moderate to severe respiratory disease in humans. Activation and dysregulation of the innate immune system play an important role in the outcome of IAV infection (*1*–*3*). Various viral factors affect innate immune dysregulation, including the RNAs that IAVs produce (*1*, *4*, *5*). Binding of IAV RNAs to host pathogen receptor retinoic acid–inducible gene I (RIG-I) is assumed to be the onset of the response to IAV infection (*6*–*8*), although other host proteins, such as interferon-γ (IFN-γ)–inducible protein 16 (IFI16), bind IAV RNAs as well (*9*). Noncanonical RNAs contribute to RIG-I activation in particular, but variation has been observed in their ability to activate RIG-I in a manner that is independent of RNA length (*10*–*12*). Once activated, RIG-I triggers the expression of IFN and proinflammatory genes, and a dysregulation of their function can affect disease severity (*3*, *13*, *14*). Presently, the molecular steps that lead from IAV RNA synthesis to IAV RNA binding by RIG-I are not understood.

**Fig. 1.**
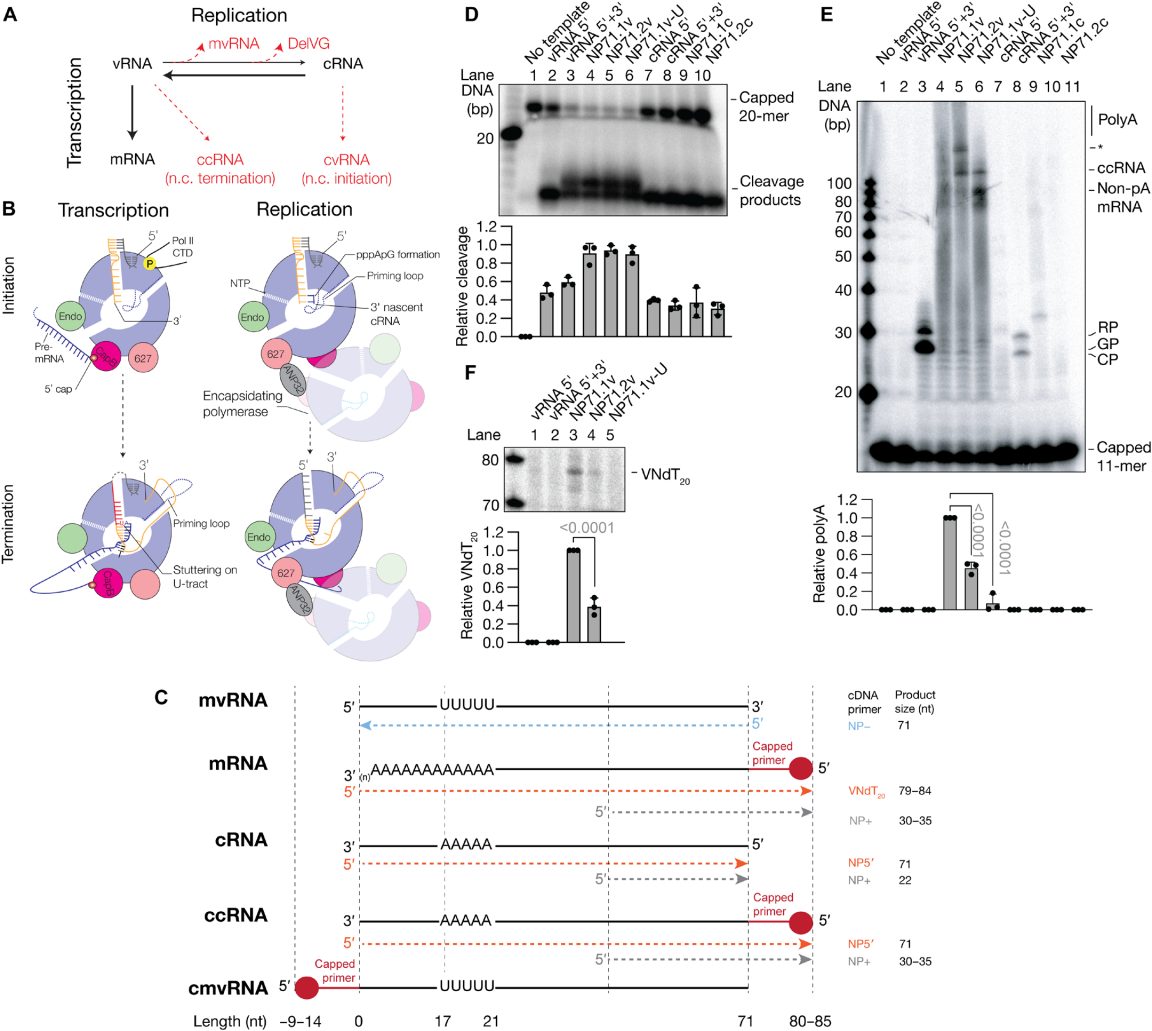
Noncanonical transcription of mvRNAs by the IAV RNA polymerase in vitro. (**A**) Schematic showing the relation between canonical and noncanonical transcription and replication during IAV infection. (**B**) Molecular differences between IAV transcription and replication. (**C**) Overview of RNA molecules produced from mvRNA templates, and the primers used to differentiate among them. (**D**) Cap-snatching of a ^32^P-labeled capped, 20-nt-long RNA primer by the IAV RNA polymerase. Cleavage reactions were analyzed by 20% denaturing PAGE. (**E**) Representative image of the extension of a ^32^P-labeled capped, 11-nt-long RNA primer by the IAV RNA polymerase. Extension reactions were analyzed by 20% denaturing PAGE. The alternative initiation products CP and GP, as well as the realignment product (RP), are indicated. Asterisk indicates an unknown signal. (**F**) Representative image of a primer extension analysis of RNA extracted from capped, 11-nt-long RNA extension reactions using VNdT_20_.

The IAV genome consists of eight segments of single-stranded, negative-sense RNA (vRNA) that contain 5′ triphosphorylated, partially complementary 5′ and 3′ promoter termini (*15*–*17*). The vRNAs exist in the context of viral nucleoproteins and a viral RNA polymerase, forming vRNPs. Upon infection, the IAV RNA polymerase first transcribes the genome segments, generating viral mRNAs (Fig. 1, A and B). This process starts with cap-snatching, which produces a 9- to 14-nt-long capped primer from host (pre)mRNAs. The capped primers are annealed to the 3′ end of the vRNA template and extended from bases C2 or G3 [generating a C-product (CP) or G-product (GP)], depending on the sequence of the primer (*18*, *19*). Realignment can take place on segments 4 to 8 when base pairing between the capped primer and the 3′ end of the vRNA template is inefficient [generating a realignment product (RP)] (*19*, *20*). When the RNA polymerase reaches a stretch of five to six U-residues (U-tract), located 16 to 17 nucleotides (nt) from the 5′ end of the vRNA template, stuttering occurs and a 3′ poly(A) tail is added to the nascent viral mRNA (*21*, *22*) (Fig. 1C). The position and length of the U-tract vary slightly among the eight IAV genome segments (fig. S1), but it is not known whether this affects the polyadenylation efficiency or length. IAV mRNAs recruit host factors UAP56, NXF1, and the TREX-2 complex to facilitate IAV mRNA export to the cytoplasm (*23*–*25*).

In contrast to transcription, IAV replication uses de novo initiation mechanisms and requires host factor ANP32 to support dimerization in cell culture (*26*, *27*). IAV replication first produces a complementary RNA (cRNA), which is a full-length copy of the vRNA template (*26*–*28*) (Fig. 1C). An encapsidating RNA polymerase binds the replicating RNA polymerase to associate NP molecules with an emerging cRNA (Fig. 1B), creating a cRNP that subsequently serves as a template for the synthesis of nascent vRNA molecules. cRNA and mRNA molecules thus vary in length and the sequence at their terminal ends as well as the protein context in which they exist (Fig. 1A). In addition to full-length molecules, IAV replication produces three noncanonical RNAs: deletion-containing viral genomes (DelVGs), mini viral RNAs (mvRNAs), and small viral RNAs (svRNAs) (*29*, *30*) (Fig. 1A). DelVGs and mvRNAs lack internal genome sequences but retain the conserved 5′ and 3′ termini of the full-length vRNA segments (*29*, *30*). Only mvRNAs are short enough (<125 nt) to be replicated and transcribed outside the context of a vRNP or cRNP (*4*, *31*, *32*), which may facilitate their detection by RIG-I and explain the correlation between their expression and the up-regulation of disease markers in infections with highly pathogenic IAV strains. svRNA are 22 to 27 nt long and only contain the 5′ terminus.

Activation of innate immune signaling by mvRNAs is not dependent on mvRNA abundance. Instead, mvRNA binding to RIG-I depends on a reduction in RNA polymerase processivity due to the formation of a template loop (t-loop) (*10*). However, how mvRNAs with t-loops trigger RIG-I activation remains unclear, as RNA polymerase dissociation is not affected by t-loops in cis in vitro (*10*), which precludes RIG-I binding. We here explored whether noncanonical or aberrant transcription of mvRNA molecules contributes to RIG-I activation. Noncanonical IAV transcripts are capped but not polyadenylated (*33*, *34*) (Fig. 1A) and referred to as capped cRNAs (ccRNAs). ccRNAs can hybridize to a complementary negative-sense RNA and activate RIG-I (*33*). We here find that mvRNAs that activate RIG-I support ccRNA formation and that ccRNAs can trigger the release of t-loop–containing mvRNAs from the RNA polymerase in a sequence-dependent manner in vitro and complement a t-loop containing mvRNA in trans in cell culture. On the basis of our findings, we propose a two-step mechanism in which sequential rounds of noncanonical replication and transcription are the start of innate immune activation by IAV RNA synthesis.

## RESULTS

### Transcription initiation occurs on positive- and negative-sense mvRNAs in vitro

We previously demonstrated that the activation of RIG-I by mvRNAs was anticorrelated with the ability of the IAV RNA polymerase to efficiently replicate an mvRNA (*10*). To investigate whether noncanonical transcription initiation might contribute to the detection of mvRNAs by RIG-I, we explored noncanonical cap-snatching on model vRNA and cRNA promoters, as well as 71-nt-long mvRNAs derived from segment 5. While noncanonical cap-snatching on cRNA promoters has been reported (*35*, *36*), it has not been studied on mvRNA-like templates. We will refer to the product of this process as a capped mvRNA [cmvRNA; the full-length equivalent would be capped vRNA (cvRNA)] (Fig. 1, A and C).

To analyze the IAV cap-snatching activity in the presence of mvRNA templates, we purified the A/WSN/33 (H1N1) RNA polymerase (abbreviated as WSN) and incubated it with a radiolabeled, capped 20-nt-long RNA in the presence of model vRNA and cRNA promoters or mvRNAs in their positive or negative sense. Specifically, we compared segment 5–derived mvRNA templates that do not (NP71.1) or do activate RIG-I (NP71.2) (*10*). As shown in Fig. 1D, the endonuclease activity was minimal when no template was added to the reaction (lane 1) and most efficient when the vRNA promoter or a vRNA-sense mvRNA was present (lanes 3 to 6). In reactions that contained both vRNA promoter termini, two cleavage products were visible that were 1 nt different in length (lanes 3 to 6). These two products were best resolved by 20% denaturing polyacrylamide gel electrophoresis (PAGE). We also observed cap cleavage activity in the presence of the cRNA promoter and the positive-sense mvRNA templates (Fig. 1D). This noncanonical cap-snatching activity on positive-sense mvRNA templates was reproducible on mvRNAs derived from segments 2, 3, 5, 6, and 8 using either a WSN RNA polymerase or an A/Vietnam/1203/2004 (H5N1) RNA polymerase (abbreviated as VN04) (fig. S2). However, the noncanonical cap-snatching activity was not dependent on the mvRNA sequence (Fig. 1D, lanes 9 and 10, and fig. S2, lanes 3 to 7), suggesting that this activity likely does not contribute to the differential RIG-I activation by mvRNAs.

To confirm that noncanonical cap-snatching was dependent on promoter recognition and not the rest of the mvRNA sequence, we generated alanine substitutions of PB1 C-terminal residues 699 to 676. Analysis of the IAV RNA polymerase transcription initiation structure showed that these PB1 residues are located on an α helix near the promoter binding site and involved in interactions with base 9G of the vRNA 3′ end or U10 of the cRNA 3′ end (fig. S3A), i.e., the first unpaired base that is different between the vRNA and cRNA promoter. Moreover, several previous observations suggested that mutation of PB1 699 to 676 could differentially affect vRNA or cRNA promoter recognition and, thus, noncanonical cap-snatching: (i) the PB1 helix undergoes a conformational change upon promoter binding (*36*); (ii) mutation of residues in the helix was shown to suppress IAV canonical cap-snatching and mRNA synthesis (*37*); and (iii) deletion of 10A (d10A) of the vRNA 5′ end, which places U10 of the vRNA 3′ end in the position of 9G (fig. S3A) and led to a reduction in mRNA synthesis (*38*). Following mutation of the PB1 subunit and purification of the mutant RNA polymerases, we observed an increase in noncanonical cap-snatching for some of the PB1 mutants relative to wild type (fig. S3B, right). Mutant S673A showed inhibited cap-snatching on both the vRNA and cRNA promoters. These results thus indicate that interactions with the promoter, rather than interactions with the downstream sequence, are important for the activation of cap-snatching and/or the dynamics of the flexible domains of the RNA polymerase in line with previous observations (*36*).

### Transcription termination of mvRNAs is sequence dependent in vitro

We next investigated whether extension of a radiolabeled, capped 11-mer primer on mvRNA templates led to noncanonical transcription termination and the production of a ccRNA. As a marker for noncanonical termination, we used a 71-nt-long mvRNA template in which the 5′ U-tract was interrupted with G-bases to prevent polyadenylation (NP71.1-U; table S1 and fig. S4A). In all reactions, we included baloxavir (BAX) to prevent cleavage of the extended capped primers by the IAV RNA polymerase (fig. S4B).

On the model vRNA promoter, the RNA polymerase efficiently extended the 3′ AG of the capped primer from the 3′ C2 or 3′ G3 of the template with or without realignment at 3′ U4 (*20*), generating products CP, GP, and RP, respectively (Fig. 1E, lane 3, and fig. S2). Extension of the capped primer also occurred on the model cRNA promoter by the WSN and the VN04 IAV RNA polymerase (Fig. 1E, lane 8, and fig. S2). This noncanonical transcription initiation activity resulted in multiple products, likely because the 3′ AG of the capped primer can be aligned in two ways opposite the 3′ UC of the cRNA template and realignment may occur on a downstream U, similar to realignment on the vRNA promoter. Transcription of the NP71.1-U template generated two main termination products, one migrating at the position of a ccRNA and the other at a position of an mRNA lacking the 3′ end entirely (Fig. 1E, lane 6), in line with our previous analyses of a segment 6 RNA with the same mutations (*33*). Analysis of reactions containing the negative-sense NP71.2 mvRNA template revealed signal accumulation at the ccRNA position and an interrupted polyadenylation signal (Fig. 1E, lane 5), whereas transcription of the NP71.1 mvRNA led to a smear of polyadenylated mRNAs. These results thus suggest that a sequence in the NP71.2 template stimulated ccRNA formation (Fig. 1E, lane 4). On the positive-sense mvRNA templates, we observed a weak extension of the capped RNA primer on all mvRNA templates tested, but did not find a difference between the NP71.1 and NP71.2 sequences (Fig. 1E, lanes 10 and 11, and fig. S2). Analysis of the activity of RNA polymerases containing PB1 mutations revealed a differential effect on capped primer extension in the presence of a negative- or positive-sense mvRNA template, particularly for PB1 N671A (fig. S3C).

To confirm the presence or absence of poly(A) tails in the in vitro transcription reactions, we performed primer extension reactions on RNA produced from the negative-sense templates with a 3′ clamped oligo-dT primer [VN(dT)_20_] as described previously (*33*). Analysis of the reaction products showed a signal in the NP71.1 reactions and a reduced signal in the NP71.2 reactions (Fig. 1F, lanes 3 and 4). No signal was observed in the presence of any of the other mvRNA templates. Overall, these results suggest that noncanonical transcription initiation and termination can occur during IAV transcription of mvRNAs in vitro and that these activities lead to the formation of ccRNA and cmvRNA molecules. The results further imply that only ccRNA formation is sequence dependent, while mcvRNA formation is not, and that an mvRNA that was previously shown to activate RIG-I (i.e., NP71.2) can trigger the formation of ccRNAs in vitro.

### mvRNAs that activate RIG-I induce ccRNA formation in cell culture

**Fig. 2.**
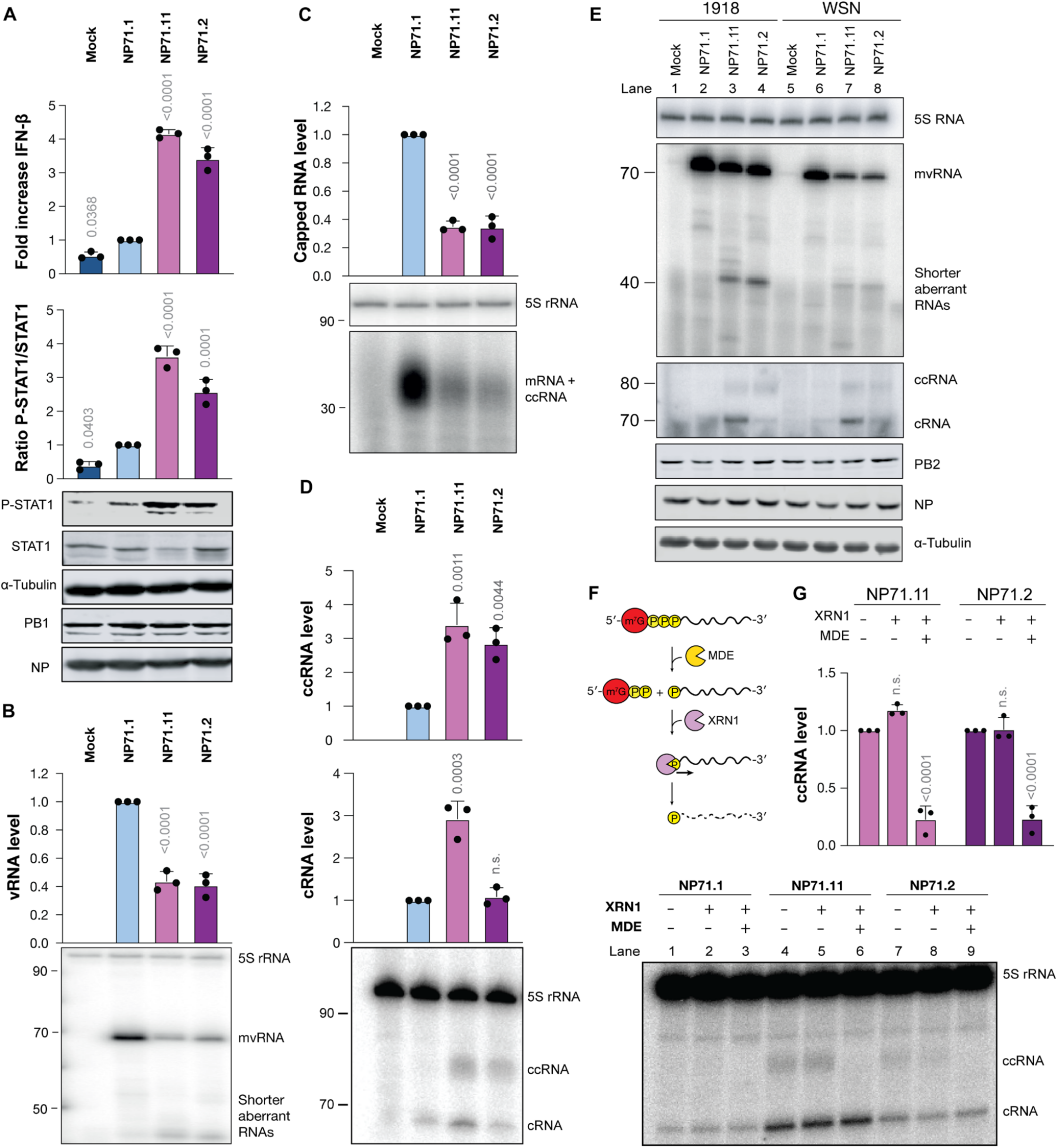
Noncanonical transcription of mvRNAs by the IAV RNA polymerase in cell culture. (**A**) Innate immune activation by IAV mvRNAs in HEK293T cells. Cells were transfected with plasmids encoding the RNA polymerase subunits, NP, a *Renilla* luciferase transfection control, a firefly-based IFN-β reporter, and the NP71.1, NP71.11, or NP71.2 mvRNAs. Twenty-four hours posttransfection, the luciferase and phosphorylated STAT1 (pSTAT1) levels were measured. PB1, NP, and tubulin protein expression was analyzed as control. (**B**) Steady-state mvRNA and 5S rRNA levels measured 24 hours posttransfection by primer extension using the NP− primer. (**C**) Steady-state total IAV capped RNA levels measured using the NP+ primer. (**D**) Steady-state cRNA and ccRNA levels measured using the NP5′ primer. (**E**) Steady-state cRNA and ccRNA levels in the presence of the WSN or A/Brevig Mission/1/1918 (H1N1) IAV RNA polymerase. (**F**) Schematic showing the removal of m^7^G from the 5′ end of viral capped RNAs using mRNA decapping enzyme (MDE), generating an RNA with a 5′ end monophosphate and the release of 7-methylguanosine disphosphate. The 5′ monosphosphate RNA is then cleaved by the exoribonuclease XRN1 in a 5′ → 3′ direction, generating ribonucleotide monophosphates. (**G**) Four micrograms of HEK293T cell RNA was subjected to enzymatic digestion using either MDE alone or a combination of MDE and XRN1. Viral RNA levels were detected using primer extension and quantified. In all figures, graphs show the mean of three independent experiments. Error bars indicate SD, and *P* values were determined using one-way ANOVA.

To investigate noncanonical transcription on mvRNAs in cell culture, human embryonic kidney (HEK) 293T cells were transfected with plasmids expressing the IAV RNA polymerase subunits, NP, and mvRNA templates NP71.1, NP71.11, NP71.2, or an empty plasmid control. After 24 hours, we extracted RNA and used various product-specific primers to analyze the steady-state RNA levels of the IAV RNA species present (Fig. 1C). In line with previous results (*10*), mvRNA templates NP71.11 and NP71.2 triggered IFN-β promoter activity in cell culture, while mvRNA template NP71.1 did not (Fig. 2A). Analysis of the total RNA using the vRNA-sense primer (NP−; Fig. 1C) showed that the NP71.11 and NP71.2 mvRNA levels were reduced and that the production of shorter aberrant products was increased (Fig. 2B, lanes 3 and 4), in agreement with our previous data (*10*). We did not see reproducible evidence for cmvRNA production from mvRNA templates in cell culture, even when we tried to increase cmvRNA production using the PB1 mutants (fig. S5). While these results do not rule out that cmvRNA or cvRNA production may occur on other IAV RNAs in cell culture, they do further indicate that cmvRNAs are not a factor in mvRNA-dependent innate immune activation.

Analysis of the total capped positive-sense RNA levels using a positive-sense–specific primer (NP+; Fig. 1C) yielded a smear of products due to the 9- to 14-nt capped primer at the 5′ end of IAV transcripts (Fig. 2C). Quantification of this signal showed that the steady-state capped RNA level was reduced by 50% for the NP71.2 and NP71.11 mvRNAs compared to NP71.1 (Fig. 2C, lanes 3 and 4). The latter result suggests that while transcription initiation between RIG-I–activating and nonactivating mvRNAs is not different in vitro, the capped RNA steady-state level is significantly different between these mvRNAs in cells.

We next analyzed the steady-state cRNA and ccRNA levels using a positive-sense primer that was specific for the 3′ end of the cRNA molecules (NP5′; Fig. 1C). As shown in Fig. 2D, we observed two bands in cells expressing mvRNAs NP71.2 and NP71.11 (Fig. 2D, lanes 3 and 4). The slower migrating of the two bands was more diffuse compared to the canonical cRNA signal and absent in NP71.1-expressing cells, suggesting that the slower-migrating signal was representative of ccRNA molecules. To confirm that this signal could also be produced by an IAV RNA polymerase other than the WSN RNA polymerase, we isolated RNA produced by the A/Brevig Mission/1/1918 (H1N1) pandemic RNA polymerase (abbreviated BM18) in transfection experiments. Analysis of the steady-state IAV RNA level demonstrated that the two products were also generated by the BM18 RNA polymerase on the NP71.2 and NP71.11 mvRNA templates (Fig. 2E).

To confirm that the slower-migrating band was a transcription product and contained a 5′ cap, RNA from WSN RNA polymerase–expressing cells was subjected to an enzymatic digestion using an mRNA decapping enzyme (MDE) or a combination of MDE and exoribonuclease 1 (Xrn1). MDE catalyzes the removal of 5′ cap-0 or cap-1 structures, resulting in the generation of an RNA with a 5′ monophosphate. This 5′ monophosphorylated RNA can be digested by Xrn1, whereas capped RNAs and 5′ triphosphorylated RNAs, such as cRNA and 5S rRNA molecules, are protected from Xrn1 digestion (Fig. 2F). As shown in Fig. 2G, in the absence of enzyme treatment or the presence of Xrn1 alone, the NP71.11 and NP71.2 reactions contained a higher–molecular weight product in addition to the cRNA signal (Fig. 2G, lanes 4, 5, 7, and 8). However, in reactions that contained both MDE and Xrn1, a reduction in the higher–molecular weight product was observed (Fig. 2G, lanes 6 and 9), while the cRNA and 5S rRNA loading control signals remained unaffected. These results, combined with the fact that the NP5′ primer was specific for the cRNA 3′ terminus, indicate (i) that transcription of the NP71.11 and NP71.2 mvRNAs produces ccRNAs and (ii) that ccRNA synthesis is dependent on the mvRNA sequence in cell culture.

### RIG-I binds mvRNAs with t-loops likely in a helicase-dependent manner

**Fig. 3.**
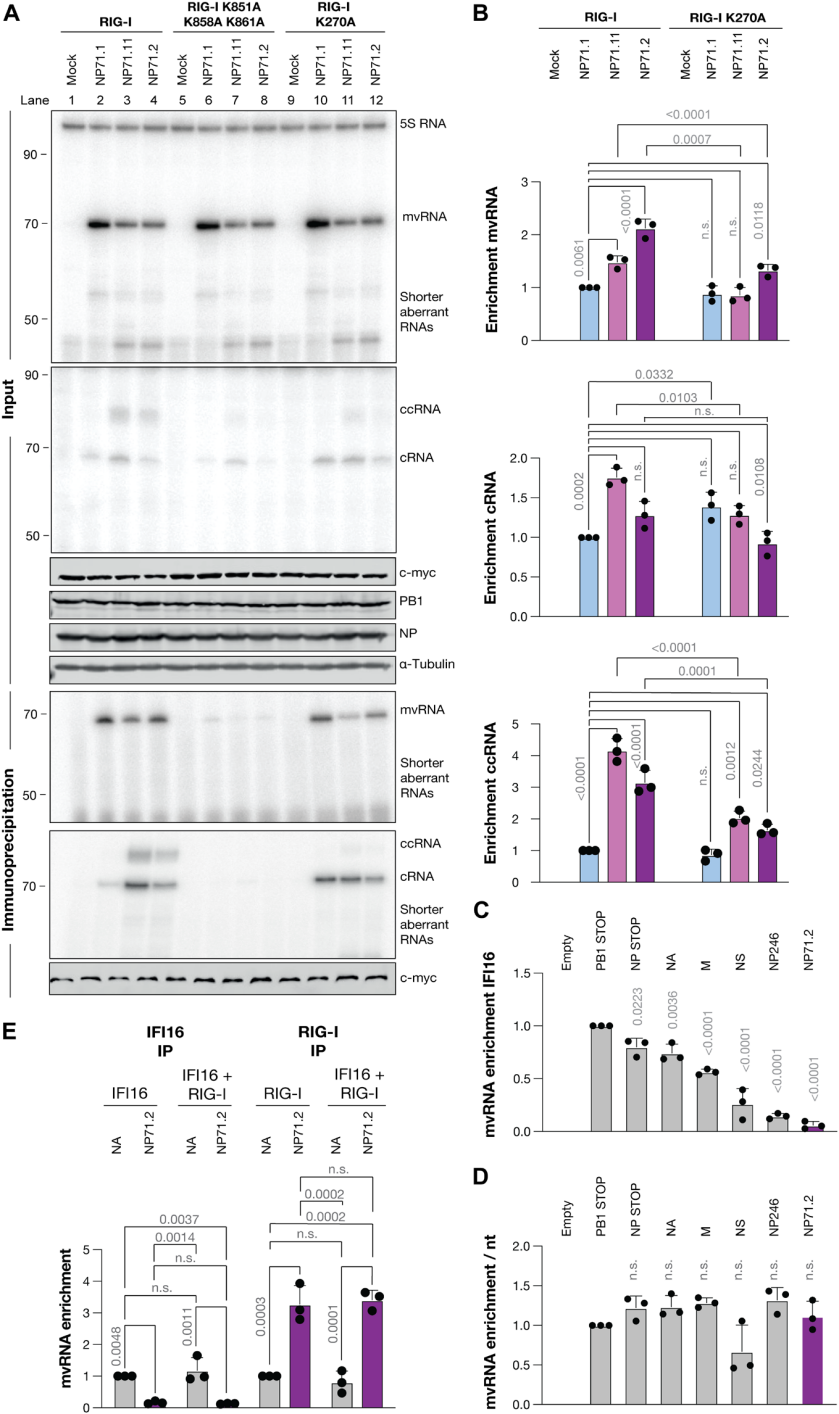
ccRNAs are enriched in RIG-I immunoprecipitation assays. (**A**) RIG-I immunoprecipitation following the expression of different mvRNAs in the presence of the WSN RNA polymerase and NP in HEK293T cells. Viral RNA levels and the 5S rRNA loading control were analyzed 24 hours posttransfection by primer extension. PB1, c-myc-RIG-I, NP, and tubulin expression were analyzed by Western blot. RNA levels following immunoprecipitation are shown in the bottom panels. (**B**) Quantification of the mvRNA, cRNA, and ccRNA levels following RIG-I immunoprecipitation. (**C**) Quantification of the mvRNA levels following IFI16 immunoprecipitation. (**D**) Normalization of vRNA enrichment by vRNA length following IFI16 immunoprecipitation. (**E**) Quantification of the mvRNA levels following RIG-I or IFI16 immunoprecipitation. In [(B) to (E)], graphs show the mean of three independent experiments. Error bars indicate SD, and *P* values were determined using one-way ANOVA.

In cells transfected with NP71.2 and NP71.11, activation of IFN-β promoter activity was RIG-I dependent (fig. S6A), in line with previous observations (*10*). IFN-β promoter activity was maintained in *MDA5*^−/−^ cells (fig. S6B), suggesting that IAV mvRNAs are detected in a dsRNA complex shorter than 500 bp (*39*). To test the ability of RIG-I to bind different mvRNAs, we transfected HEK293T cells with plasmids expressing the IAV RNA polymerase subunits, NP, and mvRNA templates NP71.1, NP71.11, or NP71.2. In addition, wild-type myc-tagged RIG-I (myc-RIG-I), a myc-RIG-I RNA binding mutant (K851A, K858A, and K861A), or a myc-RIG-I helicase mutant (R270A) was expressed. No IFN-β promoter activity was observed in cells expressing the mutant myc-RIG-I constructs (fig. S6C). After 24 hours, the steady-state RNA input and RIG-I–bound RNA levels were analyzed by primer extension (Fig. 3A and fig. S6D). As shown in Fig. 3 (A and B), in conditions in which mvRNAs NP71.11 and NP71.2 were expressed (lanes 3 and 4), a 1.5- to 2-fold enrichment in RIG-I was found compared to NP71.1 (lane 2). The differential binding of the mvRNAs to RIG-I was reduced when immunoprecipitations were performed with the RIG-I RNA binding mutant and the RIG-I helicase mutant (Fig. 3, A, lanes 5 to 12, and B). Overall, these experiments confirm that mvRNAs that activate IFN-β promoter activity are bound more efficiently to RIG-I than an mvRNA that does not activate IFN-β promoter activity. In addition, these experiments suggest that the binding of mvRNAs NP71.11 and NP71.2 to RIG-I may involve the RIG-I helicase function, which is known to play a role in RIG-I activation and template specificity (*40*).

To investigate whether the binding of NP71.2 to RIG-I is dependent on the t-loop present in the mvRNA, we also conducted RIG-I immunoprecipitations using mvRNA NP71.6, which contains two mutations that destabilize the t-loop present in the first half of NP71.2 (fig. S7A). When NP71.6 was expressed, a reduction in IFN-β promoter activity and an increase in the steady-state mvRNA level was observed relative to NP71.2 (fig. S7, B to D), confirming previous results (*10*). Analysis of the RIG-I–bound RNA showed that the NP71.2 level was enriched twofold relative to NP71.6 and NP71.1 after immunoprecipitation (fig. S7, C and E). Together, these data imply that destabilization of the t-loop influences not only the activity of the IAV RNA polymerase but also RIG-I binding, in agreement with the previously observed anticorrelation between the mvRNA replication and IFN-β signaling (*10*). Moreover, our findings indicate that ccRNA synthesis alone is not sufficient for the activation of innate immune signaling.

### mvRNA-derived ccRNAs bind RIG-I

To verify whether mvRNA-derived ccRNAs bind to RIG-I, we performed immunoprecipitations using myc-RIG-I. Analysis of the input RNA levels showed clear cRNA and ccRNA signals in reactions containing the NP71.11 and NP71.2 mvRNAs, but not the NP71.1 mvRNA (Fig. 3A, lanes 2 to 4, and fig. S6C). The steady-state cRNA levels were unchanged in the RIG-I mutant reactions compared to the wild type, except for NP71.1 in the RIG-I R270A mutant condition. In addition, we noted that the steady-state ccRNA levels were consistently lower in the RIG-I binding mutant conditions compared to wild type (Fig. 3A, lanes 6 to 8). These observations suggest that RIG-I binding affects either the formation or stability of these IAV RNAs in cell culture. After normalization of the RNA signals to the 5S rRNA loading control, we compared the input RNA levels to the RIG-I–bound RNA levels and calculated the enrichment relative to NP71.1. In conditions in which mvRNAs NP71.11 and NP71.2 were expressed, we observed a ~1.5-fold enrichment in cRNA binding to RIG-I relative to NP71.1 for NP71.11, but not for NP71.2 (Fig. 3, A, lanes 10 to 12, and B). The ccRNA signal produced by NP71.11 and NP71.2 was enriched three- to fourfold relative to NP71.1, although this difference was likely inflated by the reduced background in the NP71.1 immunoprecipitated signal. Both the cRNA and ccRNA signal were absent in immunoprecipitations with the RIG-I RNA binding mutant. In the immunoprecipitations with the RIG-I helicase mutant, the ccRNA signal was greatly reduced, while the cRNA signal was not (Fig. 3B). Together, these results suggest that there is a correlation between the binding of innate immune-activating mvRNAs to RIG-I and the binding of RIG-I to ccRNA molecules.

### IFI16 preferentially binds full-length IAV vRNAs over mvRNAs

Prior studies have shown that full-length IAV segments can bind cellular pathogen receptor IFI16 and that these interactions enhance binding of IAV RNAs to RIG-I (*9*). To exclude that IFI16 had affected our RIG-I–mvRNA binding observations, we transfected HEK293T cells with plasmids expressing flag-tagged IFI16 or a flag-tagged IFI16 RNA binding mutant (ΔHinA) in addition to the IAV RNA polymerase subunits and NP. As template for the RNA polymerase and RIG-I or IFI16, we cotransfected either an empty plasmid, a segment 6 vRNA (NA), or the segment 5–derived mvRNA NP71.2 (fig. S8). After 24 hours, the input and immunoprecipitated RNA levels were measured by primer extension. We found that the input steady-state mvRNA levels were similar between the wild-type and ΔHinA IFI16 conditions (fig. S8). Analysis of the immunoprecipitated RNA showed that the ΔHinA IFI16 mutant had not bound any RNA, whereas the wild-type IFI16 had bound the segment 6 vRNA only (fig. S8). Because none of the mvRNAs appeared to be bound by IFI16, while the full-length NA vRNA was, this suggests that IFI16 may be sensitive to the nucleic acid length, as proposed for the binding of DNA by IFI16 (*41*).

We next explored whether RNA binding by IFI16 was correlated with the length of the IAV RNA. To this end, we expressed four IAV vRNA segments of different lengths, a segment 5–derived DelVG of 246 nt (NP246) or mvRNA NP71.2 together with flag-tagged IFI16. Following immunoprecipitation, we found that vRNA segments 2 (PB1-stop; 2341 nt) and 6 (NA; 1413 nt) were strongly enriched compared to vRNA segments 7 (M; 1027 nt) and 8 (NS; 890 nt) (Fig. 3C and fig. S9). Moreover, despite the high steady-state levels of DelVG NP246 compared to the full-length vRNA segments, NP246 was not efficiently bound by IFI16. When we normalized the IFI16 binding enrichment by the RNA length, the observed differences disappeared (Fig. 3D), suggesting that IFI16 does not have an inherent preference for binding one IAV RNA over another. Instead, it appears that IFI16’s RNA binding preference is correlated with the number of nucleotides per RNA.

Because IFI16 was reported to enhance the interaction of a full-length vRNA segment with RIG-I, we considered it prudent to rule out whether the presence of IFI16 could augment or suppress the binding of mvRNAs to RIG-I. To investigate this, HEK293T cells were transfected with plasmids expressing IFI16-flag, myc-RIG-I, or both in addition to the RNA polymerase subunits, NP, and either the segment 6 vRNA, mvRNA NP71.2, or an empty plasmid (fig. S10). Next, immunoprecipitation of IFI16, RIG-I, or both was performed (fig. S10). Analysis of the input RNA showed that the steady-state RNA levels were comparable among the different conditions (fig. S10). Analysis of the IFI16-immunoprecipitated RNA showed that only segment 6 interacted substantially with IFI16, consistent with the results above (Fig. 3E). In RIG-I immunoprecipitations, both segment 6 and NP71.2 were found to interact with RIG-I, but NP71.2 exhibited an approximately threefold enrichment in RIG-I binding compared to segment 6, in line previous findings (*4*) (Fig. 3E). When RIG-I and IFI16 were coexpressed, no altered NP71.2 or segment 6 binding to RIG-I was observed, suggesting that IFI16 does not stimulate or impair mvRNA binding to RIG-I (Fig. 3E and fig. S10).

### ccRNA formation is correlated with U-tract truncation and not sufficient for IFN-β promoter activation

**Fig. 4.**
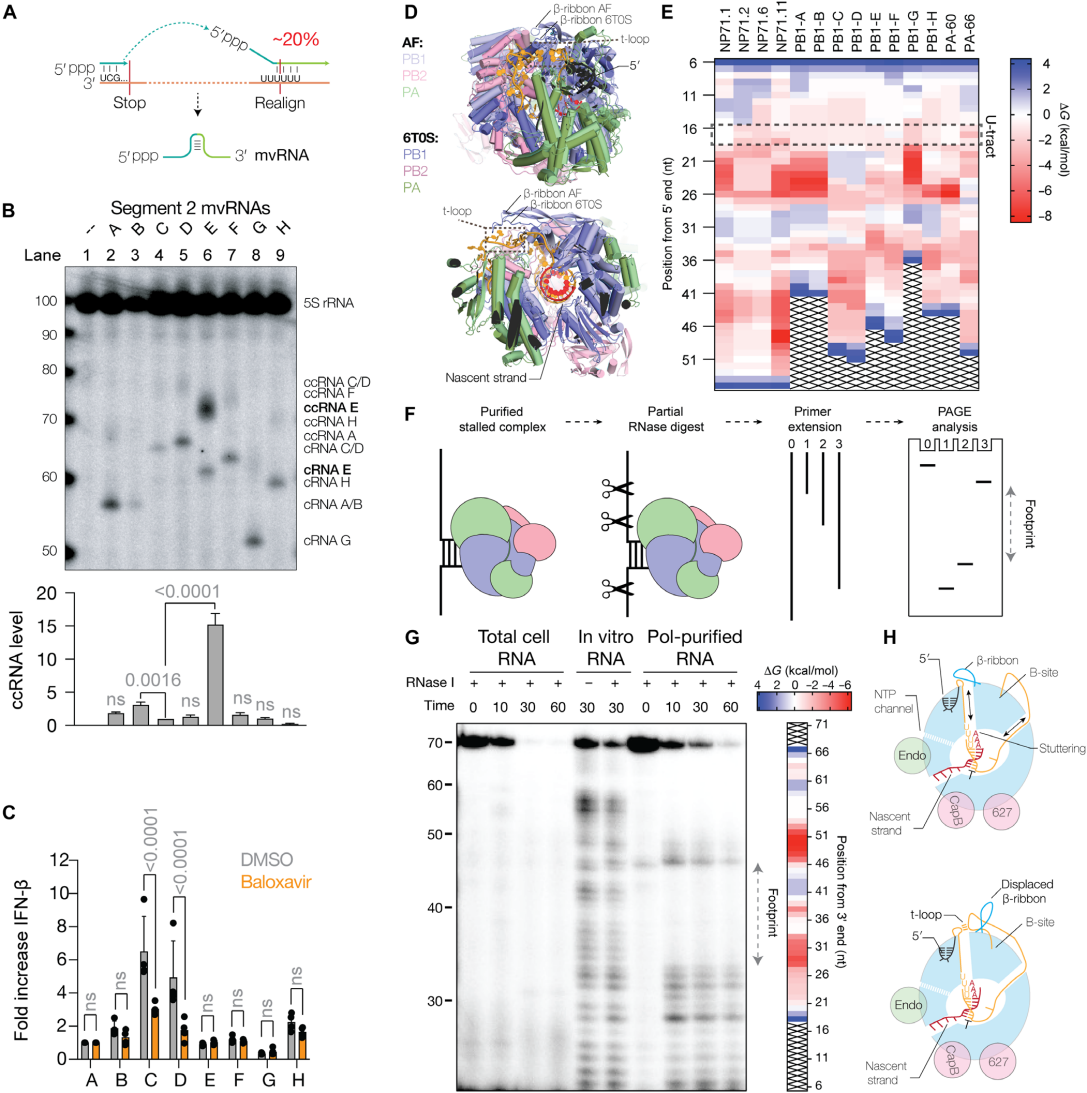
Transient RNA structures up-regulate ccRNA formation. (**A**) Schematic of the putative mechanism for mvRNA generation using template switching between the 3′ end of the vRNA template and the 5′ U-stretch. (**B**) Primer extension analysis showing ccRNA, cRNA, and mvRNA levels in HEK293T cells expressing segment 2 mvRNAs. Graphs show quantification of ccRNA level of three biological repeats. Error bars indicate SD. *P* values were calculated using one-way ANOVA relative to PB1-C. (**C**) Graph of IFN-β promoter activity measured in the presence of DMSO or baloxavir. Error bars indicate SD. *P* values were calculated using two-way ANOVA with multiple corrections. (**D**) Structural alignment of the IAV RNA polymerase in a polyadenylation state (PDB 6T0S) with an AlphaFold 3 (AF) model in which base pairing between the entering and exiting RNA shifts the position of the PB1 β-ribbon. (**E**) Heatmap showing the stability of the t-loops in the mvRNAs analyzed. The position of the t-loop is aligned relative to the 5′ terminal end of each mvRNA to better indicate position 17 of U-tract on which polyadenylation occurs. (**F**) Schematic of RNA polymerase footprinting assay. In this assay, we expressed a tandem-affinity purified (TAP)–tagged IAV RNA polymerase and an mvRNA in HEK293T cells, copurified the mvRNA with the RNA polymerase, partially digested the mvRNA parts that are not protected by the RNA polymerase, and lastly mapped the protected footprint of the RNA polymerase using primer extension. (**G**) Representative image of an RNA polymerase footprinting assay in the presence of mvRNA NP71.1. Heatmap shows calculated t-loop stability for each position of the NP71.1 mvRNA. (**H**) Model showing the role of the PB1 β-ribbon in polyadenylation and displacement of the β-ribbon by a transient RNA structure.

Naturally occurring mvRNAs are generated through template switching, leading to the formation of internal deletions (Fig. 4A). We wondered whether these internal deletions could remove residues from the U-tract. Because the U-tract is required for RNA polymerase stuttering during polyadenylation, as shown in Fig. 1 with the NP71.1-U template and by others (*21*, *22*), a shortened U-tract could provide the simplest sequence-dependent mechanism for ccRNA synthesis. Analysis of mvRNA sequences identified in A549 cells or ferret lungs infected with three different IAV strains showed that shortening of the U-tract (i.e., one to four consecutive Us) occurs in ~20% of mvRNAs (Fig. 4A and fig. S11A). It is tempting to speculate that during the synthesis of some mvRNAs, the realignment step involves the 5′ U-tract if mvRNA synthesis occurs on a negative-sense template (Fig. 4A). Alternatively, if mvRNA synthesis occurs on a positive-sense RNA template, the 3′ A-track of the cRNA may be involved. In both scenarios, realignment into the repetitive sequence would lead to truncation of the U-tract.

To confirm that ccRNA formation occurs on naturally occurring mvRNAs, we next studied six mvRNAs derived from segment 2, PB1-A to PB1-G (*10*). Alignment of the segment 2 mvRNA sequences showed that mvRNA PB1-E contained a shortened U-tract (fig. S11B). Analysis of the positive-sense RNA levels showed varying ccRNA signals and no correlation with IFN-β promoter activity (Fig. 4B). In particular, mvRNA PB1-E showed significantly increased ccRNA levels compared to the other segment 2 mvRNAs (Fig. 4B, lane 6), indicating that U-tract shortening contributes to ccRNA formation. The mvRNA levels varied in accordance with the same t-loop stability described previously (fig. S11C) (*10*) and the most suppressed mvRNAs (C and D) showed the highest IFN-β promoter activity (Fig. 4C). To test whether transcription contributed to IFN-β promoter induction by mvRNA C and D, we treated transfection reactions with PA endonuclease inhibitor BAX and observed a significant reduction in IFN-β promoter activity (Fig. 4C).

To confirm that transcription plays a role in innate immune activation by mvRNAs during infection, we overexpressed the viral RNA polymerase during WSN infection. As shown previously, this creates an imbalance between viral RNA polymerase and NP levels, and stimulates mvRNA synthesis and IFN promoter activation (*4*) (fig. S11D). The addition of BAX 2 hours postinfection to conditions in which mvRNAs were expressed reduced IFN promoter activation without significantly reducing mvRNA steady-state levels (fig. S11D). We note that these infection experiments can never be fully conclusive, because BAX may also affect the expression of viral proteins implicated in modifying the host response to the virus infection (e.g., NS1, PB1-F2, and PA-X). We are therefore cautious and conclude that the infection results indicate that there is a correlation between transcription and innate immune activation, in agreement with observations by others (*42*, *43*). Overall, the results indicate that ccRNA synthesis alone is not sufficient and a reduction in RNA polymerase processivity (i.e., reduced mvRNA replication) is needed as well. These observations are thus in line with our previous analysis showing that ccRNA molecules alone are not sufficient to activate RIG-I (*33*). Similarly, a reduction in RNA polymerase processivity on mvRNAs appears not to be sufficient for RIG-I activation and a transcriptional component is needed to trigger the induction of IFN-β promoter activity.

### The formation of ccRNAs is correlated with the presence of a terminal t-loop

We and others recently demonstrated that disruption of triple-stranded β sheet formation interrupts polyadenylation and triggers ccRNA formation (*33*, *34*). As ccRNA formation on mvRNAs was sequence dependent, we wondered whether a transient RNA structure in mvRNAs NP71.2 and NP71.11 could interrupt polyadenylation by repositioning the PB1 β-ribbon. An AlphaFold 3 (*44*) model generated with the WSN RNA polymerase amino acid sequence and an mvRNA sequence with a stabilized t-loop over the 5′ U-tract implied that the PB1 β-ribbon could be displaced upward by a t-loop (Fig. 4D). To calculate whether a similar structure could form in the mvRNA sequences tested in this study, we performed a sliding-window RNA folding analysis as described previously (*10*). The calculated Δ*G* values are presented in a heatmap with the location of the polyadenylation site highlighted with a dashed box (Fig. 4E). Our analysis of the negative-sense sequences showed that t-loop formation can potentially occur at the 5′ U-tract of all mvRNAs tested above, but not on NP71.1. To extend our comparison, we also included two segment 3 mvRNAs, one of which is known to activate RIG-I (PA-66) while the other one is not (PA-60) (*10*). We observed that the t-loop pattern at the 5′ U-tract of PA-60 was similar to NP71.1, while PA-66 has a t-loop pattern at the 5′ U-tract that was similar to NP71.2 (Fig. 4E). Analysis of the ccRNA level showed that PA-66 triggered a higher ccRNA level (fig. S12A) and IFN-β promoter activity than PA-60 (fig. S12B).

We next aimed to experimentally show that t-loops can form in cell culture using an RNA polymerase footprinting assay (Fig. 4F). Analysis of the RNA polymerase footprint on the NP71.1 mvRNA revealed an undigested region between 33 and 49 nt that was flanked by cleavage signals (Fig. 4G, lanes 8 to 10). These signals were not present when RNase I or the RNA polymerase was absent. Destabilization of the t-loop (fig. S12C) using single or double mutations changed the intensity of the footprint signal (fig. S12, D and E). The same footprint was obtained when we used MNase to digest unprotected RNA (fig. S12F). We found that the RNase-protected sequences mapped closely to the predicted t-loop sites for mvRNA template NP71.1 (Fig. 4G and fig. S12C), indicating that our sliding window analysis captures the stalling of the RNA polymerase in vitro (*10*) and in cell culture.

On the copurified NP71.2 and NP71.11 mvRNAs, no clear digestion pattern was observed (fig. S12, G and H), preventing us from confirming t-loop formation around the U-tract. In addition, we noticed that reproducibly less RNA copurified with the RNA polymerase, due to either reduced template amplification or increased template release in cell culture. While we can presently not fully rule out that the NP71.2 and NP71.11 mvRNAs contain alternative structures that could have prevented RNase digestion, the results above are overall indicative of a model in which t-loop formation near the U-tract displaces the PB1 β-ribbon and interrupts polyadenylation (Fig. 4H).

### mvRNA-derived ccRNAs activate RIG-I as part of a heteroduplex

We recently showed that ccRNAs do not induce RIG-I activation directly, but need to hybridize to a complimentary RNA, such as an mvRNA or svRNA (*33*). To confirm that this model holds true for mvRNA-derived ccRNAs, we used the PA-66 mvRNA to generate mvRNA, svRNA, and ccRNA T7 transcripts. Incubation of purified RIG-I with these RNA molecules only resulted in adenosine triphosphatase (ATPase) activity in the case of 5′ triphorphorylated mvRNA molecules, whereas the ccRNA or alkaline phosphatase-treated mvRNA molecules did not trigger ATPase activity (fig. S13). However, when we combined the ccRNA molecules with a 5′ triphosphorylated svRNA, we observed a significant increase in ATPase activity compared to ccRNA or svRNA molecules alone (fig. S13). Note that we used the short svRNA-like molecule, because the mvRNA alone already activated RIG-I and would have confounded the experiment if we had combined it with the ccRNA. Together, these results suggest that mvRNA-derived ccRNAs need to hybridize with a negative-sense molecule that contains a 5′ triphosphate to bind and activate RIG-I, in agreement with what we reported previously (*33*).

### The presence of ccRNAs can trigger mvRNA template release in vitro and complement a transcription mutant in cell culture

**Fig. 5.**
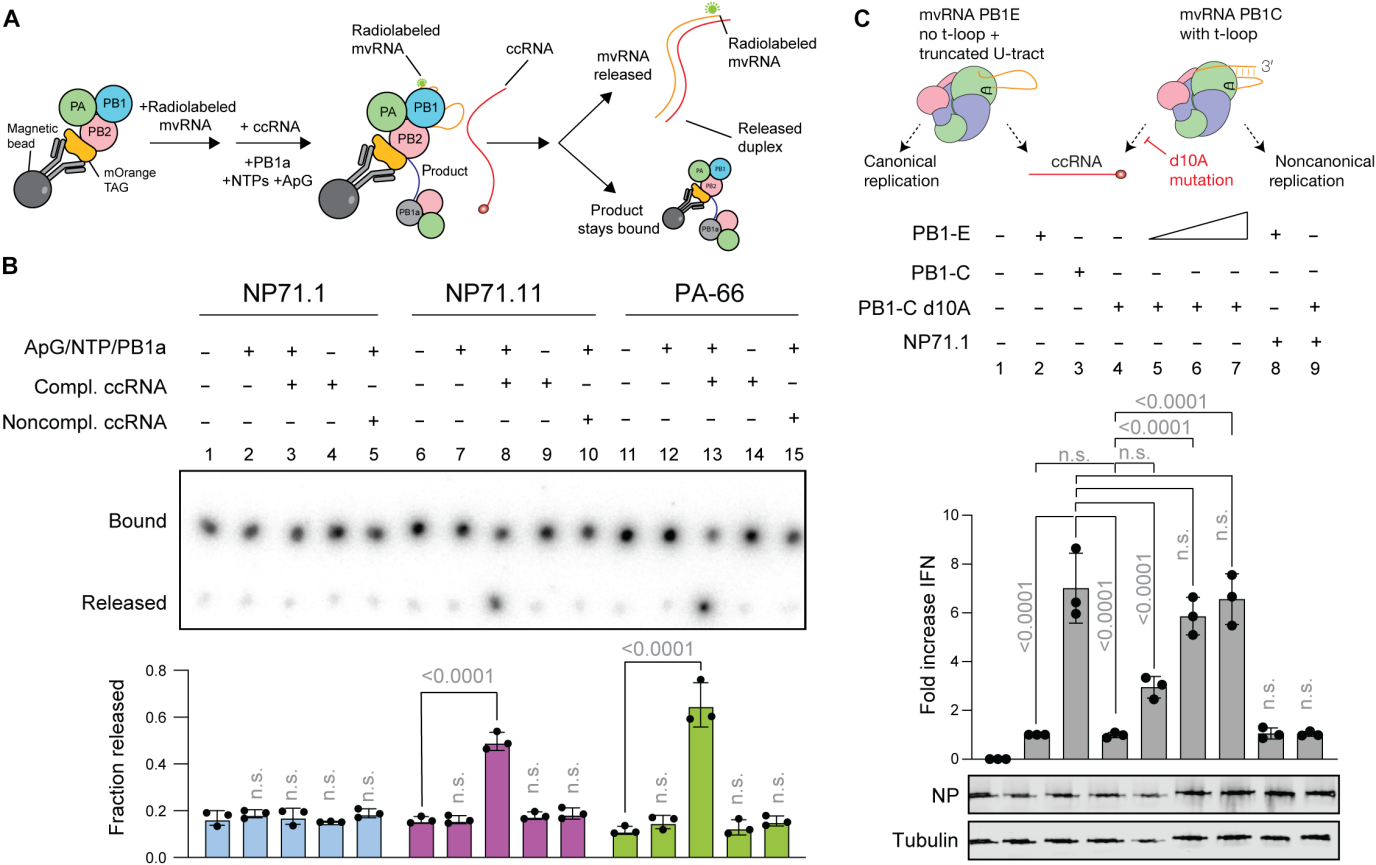
ccRNAs trigger release of mvRNA template in a sequence-dependent manner. (**A**) Schematic of assay to test the impact of ccRNA molecules on mvRNA template release during replication in vitro. Purified RNA polymerase is immobilized on magnetic beads and incubated with a radiolabeled mvRNA template. Excess template is washed away and ApG, NTPs, and an inactive RNA polymerase (PB1a) are added to start replication. A complementary ccRNA or noncomplementary ccRNA derived from a segment 6 mvRNA (NA71) is added to trigger template release. (**B**) Dot blot of RNA polymerase–bound and unbound fractions. Graph shows signal measured using radiography. (**C**) Cell-based complementation assay in which coexpression of mvRNAs leads to innate immune activation. PB1-E was titrated in combination with a fixed amount (250 ng) of PB1-C d10A at the following ratios: 1:20, 1:10, and 1.1. NP and tubulin expression were analyzed by Western blot. In [(B) and (C)], graphs show the mean of three independent experiments. Error bars indicate SD, and *P* values were determined using one-way ANOVA.

To form a duplex between a ccRNA and an mvRNA, the mvRNA template must be released by the IAV RNA polymerase. We previously showed that RNA polymerase stalling is not sufficient for mvRNA release (*10*). To test whether ccRNAs can contribute to mvRNA release in a sequence-dependent manner, we immobilized an mOrange-tagged IAV RNA polymerase on magnetic beads and added a radiolabeled mvRNA template (Fig. 5A). Excess template was removed through washing and the replication reaction started by adding NTPs, MgCl_2_, ApG, and an encapsidating inactive RNA polymerase (PB1a) as described previously (*10*). A ~2× excess of complementary ccRNA or a noncomplementary ccRNA derived from NA-71 (*33*) was added to test template release. After a 30-min incubation, we separated the free and bound fractions through centrifugation and quantified the level of template mvRNA present in each fraction using dot blotting (Fig. 5B). We found that the presence of a complementary ccRNA, but not a ccRNA that lacked complementarity, significantly increased the mvRNA signal in the unbound fraction (Fig. 5B). Next, we analyzed the unbound fraction by nondenaturing PAGE to assess potential duplex formation between the radiolabeled mvRNA and the complementary ccRNA. As shown in fig. S14, nondenaturing gel analysis showed that the mvRNA template formed a higher–molecular weight product in the presence of a ccRNA in the unbound fraction relative to the condition that lacked the ccRNA (fig. S14A).

**Fig. 6.**
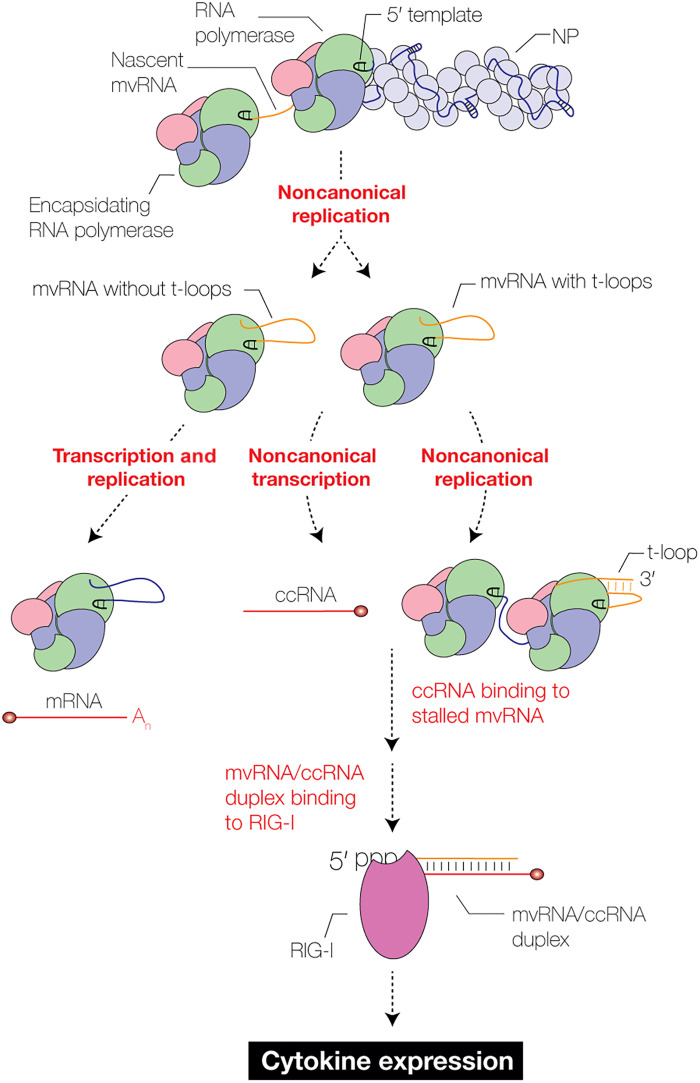
Model of a two-step mechanism that leads to innate immune activation. Model of RIG-I activation after noncanonical replication and transcription of t-loop–containing mvRNAs. Noncanonical replication produces two types of mvRNAs: mvRNAs that contain a t-loop that affects replication and/or transcription termination and mvRNAs that do not contain such a t-loop. When transcription termination is affected by t-loops, ccRNA molecules are produced. When t-loops affect replication, they can stall the RNA polymerase. ccRNAs trigger the release of mvRNAs on which the RNA polymerase has stalled, creating an RNA duplex with a triphosphate and cap. These duplexes contribute to RIG-I activation.

To investigate whether ccRNAs produced in cell culture can trigger innate immune activation through an mvRNA in trans, we performed a complementation assay. In this assay, we expressed two different mvRNAs in the presence of the viral RNA polymerase and NP (Fig. 5C): one that is not immunostimulatory but capable of producing ccRNAs through a truncation of the polyadenylation signal (PB1E; Fig. 4, B and C), and one that is immunostimulatory, but whose ability to make ccRNAs is blocked by the d10A promoter mutation that disrupts transcription (PB1C d10A; fig. S14B) (*38*). Expression of these mvRNAs alone does not lead to innate immune activation (Fig. 5C), but coexpression does in a concentration-dependent manner (Fig. 5C), suggesting that the ccRNA production by one mvRNA can make the noncanonical replication of the second mvRNA immunostimulatory. Coexpression of an mvRNA with a different sequence did not lead to complementation (Fig. 5C). These data are in line with our experiments with longer templates in which a plasmid expressed vRNA and a transfected ccRNA can activate innate immune signaling (*33*). Together, the above findings indicate that ccRNAs can contribute to the release of an mvRNA template in a sequence-dependent manner and that the noncanonical replication and transcriptional activity of the RNA polymerase on different mvRNA templates can lead to complementation in a base pair–dependent manner and activation of the innate immune response (Figs. 5, A and C, and 6).

## DISCUSSION

RIG-I is preferentially activated by 5′ tri- or diphosphorylated dsRNA molecules (*40*), but relatively few dsRNA molecules are produced during IAV infection (*7*, *45*). Instead, IAV RNA binding to RIG-I has been proposed to occur via the partially complementary 5′ and 3′ termini of each RNA. However, all IAV RNA segments and noncanonical RNAs have near-identical terminal ends, and innate immune activation by DelVGs as well as mvRNAs is sequence dependent (*10*–*12*). The precise mechanism through which some IAV RNAs become more potent inducers of innate immune signaling than other IAV RNAs remains unclear (*10*–*12*, *46*). Because aberrant positive-sense RNA virus transcripts can contribute to the activation of IFN signaling (*47*, *48*) and noncanonical IAV transcripts have been described, we here investigated the impact of noncanonical transcription on the differential detection of mvRNAs by RIG-I.

We observed that mvRNAs can trigger ccRNA formation in a sequence-dependent manner and propose that ccRNA formation occurs when the 5′ U-tract in the mvRNA is shortened during mvRNA formation or when the mvRNA contains a t-loop that dysregulates the function of the PB1 β-ribbon. This means that in addition to contributing to multibasic cleavage site emergence in the hemagglutinin-encoding gene (*49*), reducing replication (*10*), and altering genome segment stability (*50*), t-loops are implicated in a fourth IAV process. We further found that mvRNAs that (i) contain a t-loop in the first half of the RNA sequence and (ii) can trigger ccRNA synthesis are enriched in RIG-I binding experiments compared to mvRNAs that lack these characteristics, even when the latter mvRNAs were more abundant in cells due to increased replication. We confirmed that mvRNA-derived ccRNAs only activate RIG-I when a cRNA with triphosphate is present, as we showed elsewhere (*33*), and in line with the binding preference of RIG-I for a 5′-ppp and a blunt dsRNA end (*40*). Moreover, the importance of mvRNA transcription for RIG-I activation corroborates findings that link IAV transcription to innate immune activation (*42*, *43*).

RIG-I binding to RNA alone is a poor predictor of RIG-I activation. Prior research has demonstrated that RNA sampling and RIG-I activation involve multiple checkpoints, including RNA recognition, adenosine triphosphate (ATP) binding, and ATP hydrolysis (*51*–*54*). In these steps, ATP binding stabilizes RIG-I complexes, while ATP hydrolysis destabilizes them. Because RIG-I is deprived of ATP during immunoprecipitation, the experimental procedure likely freezes RIG-I in a state where the dissociation of an mvRNA cannot occur. A highly abundant mvRNA, like NP71.1, can thus be immunoprecipiated with RIG-I even when this mvRNA does not activate RIG-I. However, a low abundance mvRNA that activates RIG-I will become enriched over time. Consistent with this analysis, mutation of the RIG-I helicase/ATPase domain leads to IAV mvRNA immunoprecipitation without enrichment for IAV mvRNAs that activate innate immune signaling. In this condition, only limited RIG-I dissociation occurs and the immunoprecipitation therefore reflects abundance instead of activation. Our results agree with the above selective mechanism.

We previously showed that IAV RNA polymerase stalling does not result in the release of the RNA template or the mvRNA products in vitro. Our footprinting results confirm that once the RNA polymerase enters a strong t-loop, the RNA polymerase does not dissociate from the template RNA. We find that the presence of a ccRNA can cause template RNA release (Fig. 5B), which may lead to duplex formation (Fig. 6). We propose that duplex formation starts between the exposed part of the mvRNA (approximately 30 nt, assuming a 20-nt RNA polymerase footprint, a 10-nt promoter binding pocket, and a 10 nt t-loop duplex) and the ccRNA. Exposed RNA is present in mvRNAs bound to the RNA polymerase as they are too short for canonical NP binding. The initial binding event may next destabilize the template loop through strand displacement as observed for other RNA-RNA binding events (*55*) and/or trigger a conformational change in the RNA polymerase that causes release of the template. In tissue culture, an mvRNA that produces ccRNAs can complement an mvRNA that does not and can cause innate immune activation in trans (Fig. 5C), in line with previous transfection experiments (*33*). The duplex may be bound by RIG-I or NS1 (*56*), similar to the dsRNA structures recently identified by atomic force microscopy during IAV replication in vitro in the absence of free NP (*57*). The location where RIG-I binding to the RNA duplex occurs remains unclear. Previous fractionation assays showed that t-loop containing mvRNA templates localize to the cytoplasm and mitochondria, indicating that transport of these mvRNAs from the nucleus occurs and RIG-I binding may occur in the cytoplasm (*10*). Future experiments should specifically look into the nuclear export pathways that mvRNAs and duplexes interact with and whether cytoplasmic or nuclear RIG-I binds mvRNA-ccRNA duplexes.

Last, we observed that IFI16 does not play a role in mvRNA binding or improving the binding affinity of mvRNAs to RIG-I. Instead, we showed that the binding of IFI16 to IAV RNA is largely length dependent. Previous experiments have shown that IFI16 requires a minimum of 50 to 70 base pairs of exposed double-stranded DNA (dsDNA) to form filaments (*41*). Our results indicate that IFI16 also requires a minimal RNA length and that mvRNAs are not efficiently bound by IFI16. We therefore propose that mvRNA-ccRNA duplexes predominantly bind to RIG-I directly, without the involvement of IFI16. In contrast, full-length segments interact with both IFI16 and RIG-I, potentially engaging in competitive binding for the RNA segments, as previously suggested (*58*). While we did not test the impact of mvRNA-ccRNA duplex formation on IFI16 binding, we expect this not to have been a differentiating factor because IFI16 can bind dsDNA. Overall, our results indicate that different IAV RNA species interact with host innate immune receptors in different steps or different pathways and that noncanonical transcription as well as modulation of RNA polymerase processivity during replication is required for activation of the innate immune response by mvRNAs (Fig. 6).

## MATERIALS AND METHODS

### Cells and transfection

HEK293T and Madin-Darby canine kidney (MDCK) cells were obtained from the American Type Culture Collection (ATCC) and confirmed to be free of mycoplasma. HEK293, HEK293 *RIG-I*^−/−^, and HEK293 *MDA5*^−/−^ cells were a gift from J. Rehwinkel (Oxford University) and have been described previously (*59*). All cells were grown in Dulbecco’s modified Eagle’s medium (DMEM) high glucose with l-glutamine and pyruvate (Gendepot), supplemented with 10% fetal bovine serum (FBS HI, Gibco Life Technologies). The cells were maintained at 37°C and 5% CO_2_. Cells were counted with a Countess 3 automated cell counter (Invitrogen) before experiments.

### Plasmids

PB1 mutations described in this study were introduced using site-directed mutagenesis using primers listed in table S2. The pPolI plasmids encoding A/WSN/33 genome segments were previously described in (*60*); the pPolI NP246 plasmid was described in (*4*); the pcDNA3 plasmids encoding wild-type A/WSN/33 proteins were described in (*61*); the pcDNA3 plasmid encoding PB1 D445A/D446A (PB1a) mutant was described in (*62*); the pcDNA3 plasmid encoding myc-RIG-I wild type and mutants was described in (*4*, *33*); the pcDNA3 plasmid encoding tandem-affinity purified (TAP)–tagged PB2 was described in (*63*); the plasmid expressing firefly luciferase under IFN-β promoter [pIFD(−116)lucter] and pcDNA3 encoding *Renilla* luciferase were described in (*10*); pcDNA3 plasmids encoding flag-tagged IFI16 wild type and mutant were described previously (*64*) and obtained from Addgene (plasmid numbers 35064 and 35062).

### Transfections

The RNP reconstitution assays were performed as described previously (*65*). Briefly, confluent cells were trypsinized, washed with phosphate-buffered saline (PBS), and resuspended in 10 ml of DMEM/10% FBS. Cells were counted and 4 × 10^5^ HEK293T cells were seeded in a 24-well plate, and transfected in suspension with 250 ng of the WT plasmids pcDNA3-NP, pcDNA3-PA, pcDNA3-PB2, WT or mutated pcDNA3-PB1, and a pPolI plasmid encoding a vRNA full-length or truncated vRNA/cRNA templates (table S2) using Lipofectamine 2000 (Invitrogen) according to the manufacturer’s instructions. Next, 500 μl of DMEM/10% FBS containing 1% penicillin/streptomycin (Gibco) was added to each well. The cells were incubated for 24 hours, resuspended in 1 ml of PBS, and split into three equal parts. Cell suspension (500 μl) was used for RNA isolation, 250 μl for Western blot analysis and another 250 μl for the IFN-β promoter expression assay. Cells were pelleted at 700*g* for 5 min and analyzed.

### IFN-β promoter

For IFN-β promoter activity assays, 100 ng per well of a plasmid expressing firefly luciferase from the IFN-β promoter and 10 ng per well of a plasmid expressing *Renilla* luciferase from the CMV promoter were transfected alongside IAV expression plasmids. Twenty-four hours after transfection, cells were collected, and a quarter of each pellet was resuspended in 50 μl of PBS. Next, 25 μl of cell suspension was transferred in duplicate to a white 96-well plate (Greiner Bio-One) and mixed with 25 μl of substrate and lysis buffer. The firefly luciferase and *Renilla* luciferase activities were measured using a Dual-Glo Luciferase Assay System (Promega) according to the manufacturer’s protocol. The signals were measured on a Synergy LX (Bio-TEK) plate reader. The signal of nontransfected cells was used as background and subtracted from firefly luciferase and *Renilla* luciferase signals. Firefly luciferase signal was divided by the *Renilla* luciferase signal to normalize the firefly luciferase signal for transfection efficiency. As transfection controls for IFN-β promoter activation by RIG-I or MDA5, we used high– and low–molecular weight dsRNAs (InvivoGen).

### Virus and infection

A/WSN/33 (H1N1) was rescued using the 12-plasmid system (*60*). Virus stocks were grown on MDCK cells at an multiplicity of infection (MOI) of 0.01. For BAX experiments, HEK293T cells were transfected (250 ng) with pcDNA3 plasmids expressing the A/WSN/33 (H1N1) RNA polymerase or an empty pcDNA3 plasmid, 100 ng of IFN-β reporter plasmid, and 10 ng of *Renilla* transfection control plasmid. After 24 hours, the cells were infected or mock infected with A/WSN/33 (H1N1) at an MOI of 3. After 2 hours, BAX was added to a final concentration of 30 nM in DMEM/0.5% FBS. Eight hours postinfection, the cells were harvested and the firefly and *Renilla* luciferase signals were measured. RNA was extracted for mvRNA RT-PCR as described previously (*4*).

### RIG-I activity assay

Recombinant RIG-I was purified as described in (*66*). For ATPase assays, 0.5 μM RIG-I was incubated with 0.1 μM [γ-^32^P]ATP and 10 ng of template RNA as previously described (*33*). [γ-^32^P]ATP and ^32^P_i_ were resolved using polyethylenimine (PEI)-cellulose thin layer chromatography (TLC) plates (Sigma-Aldrich). After wrapping the TLC plates in plastic foil, the radioactive signals were detected using phosphorimaging.

### RNA extraction

Cell pellets were resuspended in 250 μl of TRI Reagent (MRC) and mixed in a 5:1 ratio with chloroform by vortexing. The suspension was centrifuged for 15 min at 10,000*g* and 4°C. The aqueous phase (approximately 150 μl) containing the RNA was transferred to a fresh tube, mixed with 1.5 μl of GlycoBlue TM Coprecipitant (Thermo Fisher Scientific) and 1 volume (approximately 150 μl) of isopropanol. After mixing by inversion, the RNA was centrifuged for 15 min at 17,000*g* and 4°C. The RNA pellet was subsequently washed with 75% ethanol and centrifuged for 5 min at 17,000*g* and 4°C. Residual ethanol was removed by a further 10-s centrifugation step. Last, RNA pellets were resuspended in 10 μl of RNase-free water and the RNA concentration was measured by the NanoDrop spectrophotometer (Thermo Fisher Scientific).

### Primer labeling

DNA oligonucleotide (1 μM; table S3) was incubated with 10 U of T4 polynucleotide kinase (NEB), 1× buffer T4 polynucleotide kinase, and 1 μl of [γ-^32^P]ATP (6000 Ci/mmol; Perkin Elmer) in a total reaction volume of 10 μl at 37°C for 1 hour. Unincorporated radioactive label was removed using an Oligo Clean & Concentrator Kit (Zymo Research) according to the manufacturer′s instructions, and radiolabeled oligonucleotides were eluted in 30 μl of RNase-free water.

### Primer extension

For quantitative primer extensions, reverse transcription was carried out using SuperScript III reverse transcriptase (Thermo Fisher Scientific) with ^32^P-labeled oligonucleotides (table S3 and Fig. 1) that were complementary to IAV vRNA and ribosomal 5S rRNA (5S rRNA). First, 2 to 4 μl of total RNA was annealed to 1 μl of the radioactive primer mix (0.25 μl of 0.3 μM ^32^P-primer, 0.05 μl of 0.3 μM ^32^P-5S_100 primer, 0.45 μl of 10 μM unlabeled 5S_100 primer, and 0.25 μl of water) incubated at 95°C for 2 min and immediately cooled on ice. Reverse transcription was conducted in a 10-μl reaction volume using 1× First-strand buffer, 50 U of SuperScript III Reverse Transcriptase (Invitrogen), 10 mM dithiothreitol (DTT), 10 U of RNase inhibitor (APExBio), and 0.5 mM dNTPs by incubating RNA samples at 50°C for 1 hour. Ten microliters of formamide loading dye (90% formamide, 10 mM EDTA, 0.25% bromophenol blue, and 0.25% xylene cyanol FF) was added and the samples were denatured at 95°C for 2 min. The ^32^P-labeled cDNA products were resolved by 6 or 12% denaturing PAGE [19:1 acrylamide/bisacrylamide (Bio-Rad), 1× tris borate buffer (Growcells), 7 M urea, 0.1% ammonium persulfate (APS), and 0.1% tetramethylethylenediamine (TEMED)] for 1 hour at 35 W and 2000 V. The 6% denaturing PAGE gels were dried on chromatography paper grade 1 (Cytiva) using a gel dryer (Model 583, Bio-Rad) at 80°C, for 1 hour. The radiolabeled signals were measured using phosphorimaging on a typhoon scanner (GE Healthcare). The 5S rRNA signal was used to normalize the viral RNA levels.

### Decapping

To assess the presence of an m^7^G cap on the extended cRNA products, 4 μg of total cellular RNA was incubated in a 40-μl reaction mix with 1× MDE reaction buffer, 15 U of MDE (NEB), and 40 U of RNase inhibitor (APExBio) at 37°C for 2 hours. RNA was purified using an RNA clean and concentrator 5 kit (Zymo Research) and eluted in 15 μl of RNase free water. In a 50-μl reaction mix, 15 μl of the eluted RNA was further digested in the presence of 1× NEBuffer 3, 50 U of RNase inhibitor (APExBio), and 5 U of XRN-1 (NEB). These reactions were incubated at 37°C for 2 hours. Last, the RNA was purified using a RNA clean and concentrator 5 kit (Zymo Research) and eluted in 10 μl for primer extension analysis.

### Western blot

Protein samples were resuspended in 5× Laemmli buffer (for 5×; 300 mM tris-HCl, pH 6.8, 10% SDS, 50% glycerol, 0.05% bromophenol blue, and 100 mM DTT) and denatured at 95°C for 5 min. Insoluble debris was pelleted through centrifugation at 17,000*g* for 5 min. The SDS-PAGE gels consisted of two parts: a bottom 8% resolving gel [375 mM tris-HCl, pH 8.8, 0.1% SDS, 8% acrylamide/bis 37.5:1 (Bio-Rad), 0.1% APS, and 0.1% TEMED] and a 3.2% top stacking gel [125 mM tris-HCl, pH 6.8, 0.1% SDS, 3.2% acrylamide/bis 37.5:1 (Bio-Rad), 0.1% APS, and 0.1% TEMED]. Samples were run at 100 V in 1× tris-glycine SDS running buffer (Invitrogen). Proteins were transferred to a Protan 0.45 μM (Cytiva) at 25 V, 1 A for 25 min using transfer buffer (25 mM tris, 192 mM glycine, and 20% ethanol), Whatman gel blotting paper (Cytiva), and a Trans-Blot Turbo transfer system (Bio-Rad). The membranes were first incubated with blocking buffer (PBS-1×, 5% bovine serum albumin, and 0.1% Tween 20) for 1 hour. The membranes were incubated overnight at 4°C with primary antibodies (table S3) diluted in blocking buffer. Before adding secondary antibodies at 4°C for 2 hours (Table), the membranes were washed three times with PBS 0.05% Tween 20 for 10 min. Membranes were washed again three times before the signals were measured on an Odyssey CLx (LI-COR).

### RNA polymerase purification

Twenty-four hours before transfection, 5.5 × 10^6^ HEK293T cells were plated in a 10-cm dish. The cells were transfected with 4 μg of pcDNA3 plasmids expressing the PB1 subunit, the PA subunit, and a TAP-tagged PB2 subunit, using PEI (1 mg/ml) with a 1:5 DNA:PEI (μg:μl) ratio. Forty-eight hours after transfection, the cells were washed two times with cold PBS, lysed with 1 ml of lysis buffer [50 mM Hepes, pH 8, 200 mM NaCl, 25% glycerol (Sigma #G5516-1L), 2% Tween 20 (RPI #9005-64-5), 1 mM β-mercaptoethanol (Bio-Rad), and 1× EDTA-free Protease inhibitor cocktail (Roche)] directly in the dish and harvested. The cells were further lysed by rotation for 1 hour at 4°C before being sonicated. The lysate was centrifuged at 17,000*g* for 15 min at 4°C. The TAP-tagged RNA polymerase was bound to 50 μl of immunoglobulin G (IgG) Sepharose beads (GE Healthcare) that were prewashed three times in binding buffer (50 mM Hepes, pH 8, 200 mM NaCl, 25% glycerol, and 2% Tween 20). After 24 hours of continuous rotation at 4°C, the beads were washed three times in binding buffer and one time in cleavage buffer (50 mM Hepes, pH 7.5, 200 mM NaCl, 25% glycerol, 0.5% Tween, and 1 mM DTT). Each wash was followed by 10 min rotation at 4°C. Last, tobacco etch virus (TEV) protease cleavage was conducted with 15 U of AcTEV protease (Invitrogen, #12575-015) in 250 μl of cleavage buffer overnight. The beads were separated from the cleaved RNA polymerase by centrifugation at 500*g* for 1 min and the partially purified RNA polymerase was analyzed through SDS-PAGE and silver staining using a SilverXpress kit (Invitrogen).

### RNA polymerase footprinting

Transfections were performed as described above for the RNA polymerase purification with minor modifications. The cells were transfected with 4 μg of pcDNA3 plasmids expressing the wild-type PB1 and PA subunits, a TAP-tagged PB2 subunit, and a pPolI plasmid expressing an mvRNA template using a 1:5 DNA:PEI (μg:μl) ratio. Forty-eight hours after transfection, the cells were harvested in cold PBS, washed once with cold PBS, and lysed with 1 ml of EDTA lysis buffer (50 mM Hepes, pH 8, 5 mM EDTA, 200 mM NaCl, 25% glycerol, 2% Tween 20, 1 mM β-mercaptoethanol, and 1× EDTA-free Protease inhibitor cocktail). Lysis was continued for 1 hour at 4°C and completed using sonication. The lysate was cleared through centrifugation at 17,000*g* for 15 min at 4°C. The TAP-tagged RNA polymerase bound to 50 μl of IgG Sepharose beads (GE Healthcare) that were prewashed three times in lysis buffer for 2 hours at 4°C under continuous rotation. To remove background RNA, the beads were washed three times with lysis buffer. Last, RNase I (Thermo Fisher Scientific #EN0602) was added into the lysis buffer using the amounts indicated for the experiments, or micrococcoal nuclease (Thermo Fisher Scientific #EN0181) was added in the manufacturer’s reaction buffer as indicated for the experiments. Undigested RNA was extracted using 250 μl of TRI Reagent (MRC) as described above.

### Immunoprecipitation

Twenty-four hours before transfection, 5.5 × 10^6^ HEK293T cells were plated in a 10-cm dish. The cells were transfected with 4 μg of pcDNA plasmids expressing the PB1 subunit, the PA subunit, and a PB2 subunit and 4 μg of pcDNA c-myc RIG-I or/and pcDNA c-myc IFI-16 and mvRNA or full-length segments encoded by a pPol-I plasmid, using PEI (1 mg/ml; Sigma-Aldrich) with a ratio 1:5 DNA:PEI. Forty-eight hours after transfection, the cells were washed two times with cold PBS, lysed with 600 μl of lysis buffer [10 mM tris-HCl, pH 7.5, 200 mM NaCl, 0.5% NP40, and 1× EDTA-free Protease inhibitor cocktail (Roche)] directly in the dish, harvested with a cell scraper, and incubated for 1 hour at 4°C on a rotation wheel before being sonicated. The lysate was centrifuged at 17,000*g* for 15 min at 4°C. The lysates were further diluted with 900 μl of dilution buffer [10 mM tris-HCl, pH 7.5, 200 mM NaCl, and 1× EDTA-free protease inhibitor cocktail (Roche)]. The c-myc-RIG-I or Flag-IFI16 was bound to 25 μl of Myc-TRAP agarose (Chromotek) or Fab-TRAP agarose (Chromotek) that was prewashed three times in wash buffer (10 mM tris-HCl, pH 7.5, 200 mM NaCl, and 0.05% NP40). Each wash is followed by 10 min of rotation at 4°C. Beads were resuspended in 100 μl of wash buffer. Eighty microliters of this resuspension was used for RNA extraction and 20 μl was used for protein analysis through SDS-PAGE and Western blot analysis.

### Primer capping

The capping of RNA primers was conducted as described previously (*27*). Briefly, 1 μM 20-nt-long RNA (ppAAUCUAUAAUAGCAUUAUCC; Chemgenes) or 11-nt-long RNA (ppGAAUACUCAAG) was capped with a radiolabeled cap-1 structure in a 10-μl reaction containing 0.15 μM [α-^32^P]guanosine-5′-triphosphate (GTP) (3000 Ci mmol^−1^; Perkin-Elmer), 1× Capping buffer (NEB), 0.8 mM *S*-adenosylmethionine (SAM) (NEB), 5 U of vaccina virus capping system (NEB, M2080S), and 25 U of mRNA Cap 2′ *O*-methyltransferase (NEB, M0366S) for 1 hour at 37°C and for 4 min at 70°C. Unincorporated radiolabel was removed with the Oligo Clean & Concentrator Kit (Zymo Research) according to the manufacturer′s instructions, and radiolabeled RNAs were eluted in 30 μl of RNase-free water.

### Cap cleavage

The cleavage of RNA primers by the IAV RdRp was conducted as described previously (*27*). To test the endonuclease activity of the purified IAV RdRp in a 4-μl reaction, ^32^P-labeled and capped 20-nt-long RNA primer was incubated with 1 mM DTT, 0.7 μM template (table S1), 5 mM MgCl_2_, RNase inhibitor (1 U/μl; APExBio), 50 mM Hepes (pH 7.5), 200 mM NaCl, 25% glycerol, 0.5% Tween 20, and ∼2 ng of RNA polymerase μl^−1^. The reaction mixtures were incubated for 1 hour at 30°C and stopped with formamide loading dye, denatured at 95°C for 2 min, and analyzed by 20% denaturing PAGE. The capped RNA cleavage products were visualized by phosphorimaging.

### Capped primer extension

To measure the extension and polyadenylation of mvRNA templates (table S1) by the IAV RNA polymerase, we followed the protocol described previously (*33*). Briefly, ∼2 ng of RNA polymerase μl^−1^ was incubated for 10 min at 30°C in the presence 1 mM DTT, 5 mM MgCl_2_, RNase inhibitor (1 U/μl; APExBio), 50 mM Hepes (pH 7.5), 200 mM NaCl, 25% glycerol, 0.5% Tween 20, and 15 μM BAX (Chemgenes). Next, the following components were added to the RNA polymerase mixture: 500 μM uridine-5′-triphosphate (UTP), 500 μM ATP, 500 μM cytosine-5′-triphosphate (CTP), 500 μM GTP, ^32^P-labeled and capped 11 nucleotide-long RNA primer, and 0.7 μM mvRNA template (table S1). The reaction mixtures were incubated for 1 hour at 30°C and stopped with 4 μl of formamide loading dye. Samples were subsequently denatured at 95°C for 2 min and analyzed by 12% denaturing PAGE. Reaction products were visualized by phosphorimaging on a Typhoon scanner (GE Healthcare).

### T-loop and sequence analysis

The t-loop analysis was performed using a Python script as described previously. The distance between the entry and exit channels was set to 20 nt of the template sequence and the increment of the sliding window was set to 1 nt. The formation of secondary structures was measured by computing the Δ*G* between 6 nt upstream and 6 nt downstream of the location of the RNA polymerase. To analyze the length of U-tracts in mvRNA sequences, mvRNA sequences were retrieved from publicly available datasets described in (*10*).

### Statistical analysis

Experiments were repeated as indicated in each figure. Error bars in each figure indicate SD. Data were plotted using Prism 10 (GraphPad) and analyzed using one-way analysis of variance (ANOVA) with Dunnett’s multiple comparisons test or two-way ANOVA where indicated in the figure legends.
